# Comparative histological characteristics of the nasal cavity of Beagle dogs and rabbits

**DOI:** 10.4142/jvs.25354

**Published:** 2026-07-06

**Authors:** Seongmo Yang, Heesu Ji, Sanggu Kim, Soochong Kim

**Affiliations:** 1 Laboratory of Veterinary Pathology, College of Veterinary Medicine, Chungbuk National University, Cheongju 28644, Korea.; 2 Cancer Research Center, Chungbuk National University, Cheongju 28644, Korea.

**Keywords:** Turbinates, histopathology, standardization, anatomical landmarks, toxicity

## Abstract

**Importance:**

The nasal turbinate is a structurally complex organ essential for air conditioning and defense against inhaled toxicants. Despite its importance, standardized histological criteria for Beagle nasal turbinates have not been fully established, limiting the reproducibility and comparability of toxicological and pathological evaluations.

**Objective:**

To characterize region-specific histological features of the Beagle nasal turbinates using anatomically defined anterior, middle, posterior, and olfactory regions.

**Methods:**

Nasal cavities from five 8-month-old Beagle dogs and five 10-week-old New Zealand White rabbits were sampled at four anatomically defined transverse levels corresponding to the anterior (last upper incisor teeth), middle (upper canine teeth), posterior (third premolar teeth), and olfactory (fourth premolar teeth) regions, using reproducible dental and cranial landmarks. Hematoxylin and eosin and periodic acid–Schiff staining were performed to evaluate epithelial types, submucosal structures, and goblet cell distribution.

**Results:**

Gross transverse sections revealed clear anatomical differences between Beagles and rabbits, particularly in turbinate complexity. In the anterior region, Beagles were entirely lined by transitional epithelium, whereas rabbits had a mixed layer of stratified squamous epithelium. In the middle region, Beagles showed a broader distribution of respiratory epithelium with abundant goblet cells. In the posterior and olfactory regions, where olfactory epithelium was mainly distributed, Beagles showed more well-developed and significantly thicker olfactory epithelium compared to rabbits; mean difference 39.889 μm (95% CI, 11.079–68.698), *p* = 0.0138.

**Conclusions and Relevance:**

This study is the first to propose standardized, region-specific histological criteria for Beagle nasal turbinates, supported by quantitative analysis. By presenting its unique histological structures and traits, this study provides a reproducible reference framework for Beagle nasal cavity histology.

## INTRODUCTION

Inhalation toxicity studies have traditionally focused on the lower respiratory tract because of its central role in gas exchange and vulnerability to toxic injury [1,2]. However, the nasal cavity is the primary barrier to inhaled toxicants and supplies essential mucosal defense [3,4]. The nasal mucosa comprises squamous, transitional, respiratory, and olfactory epithelia, each supporting distinct functions including mucociliary clearance, odor detection, and host defense. Squamous epithelium is confined to the entrance of the nasal opening, and it is continued by transitional epithelium, which is a non-ciliated cuboidal to columnar epithelium prior to the caudal respiratory epithelium [5]. Therefore, region-specific evaluation of the nasal cavity enables precise identification of localized effects of inhaled substances [1,6].

Guidance from the International Council for Harmonization and the U.S. Food and Drug Administration recommends using non-rodent models, such as rabbits and Beagles, in inhalation toxicity studies to better approximate human respiratory physiology [1,7]. Beagles have a dolichocephalic skull with a long nasal passage and complex turbinate anatomy, which together provide an expanded mucosal surface area and enable detailed assessment of localized tissue responses [8,9]. The longer nasal passage increases the retention time of inhaled substances, making the Beagle a suitable model for upper airway toxicity and for chronic exposure [6,10].

Rabbits are often chosen as an experimental model because of their established role in inhalation toxicity studies and existing histological standards [6,10]. Rodents and rabbits have been extensively studied for their nasal histology and responses to toxicants. It is well known that rodents and rabbits exhibit substantial differences in epithelial distribution, mucosal gland density, and olfactory region organization [11,12]. Such differences may influence toxicant deposition and tissue responses, making it difficult to directly extrapolate the data of one species to another. Despite their frequent use in regulatory toxicology, comprehensive and standardized histological characterization of Beagle nasal turbinates has not yet been established. Moreover, reference-based sectioning criteria for the Beagle nasal cavity are currently lacking. Previous reference data have primarily focused on rodents and rabbits, leaving a critical gap in Beagle-specific information, undermining reproducibility and consistency in toxicological and pathological evaluations [12,13].

Considering the dolichocephalic skull structure and highly developed olfactory capacity of the Beagle, we hypothesized that their nasal turbinates would exhibit greater structural complexity compared to those of rabbits. Despite this intricate anatomical structure, Beagle nasal turbinates would exhibit consistent and reproducible histological landmarks. Therefore, we aimed to establish standardized, region-specific histological criteria of Beagle nasal turbinates. These findings provide essential baseline data for inhalation toxicology and pathology, and are expected to improve reproducibility, comparability, and interpretability in future studies utilizing Beagle models.

## METHODS

### Study design

This study was designed as a cross-sectional comparative study. All animal experiments were performed at Biotoxtech (Korea), a Good Laboratory Practice-certified facility authorized to conduct non-clinical studies. Experimental protocols were reviewed and approved by the Institutional Animal Care and Use Committee (IACUC Approval No. 241100000053 and 250300000074).

### Animals

Five 10-week-old New Zealand White rabbits (four females and one male; weight: 2.00 ± 0.10 kg) were used as a comparative reference group. Euthanasia was performed using pentobarbital sodium (150 mg/kg, intravenous [IV]; Hanlim Pharm, Korea), followed by exsanguination via the abdominal aorta. The study was conducted under the internal study number BKRC2500312.

For cross-species comparison, five 8-month-old Beagle dogs (five females; weight: 6.50 ± 0.35 kg) were obtained from Jiangsu Johnsen Bioresource (China). Beagles were selected because of their frequent use in inhalation toxicity studies and the availability of historical control data [1]. Euthanasia was performed using pentobarbital sodium (150 mg/kg, IV), followed by exsanguination via the axillary artery. The study was conducted under the internal study number BKRC2401261.

The age of each species was selected to match the adolescent developmental stage to control for developmental differences between rabbits and Beagles [14,15].

### Region-based sampling

**Fig. 1:**
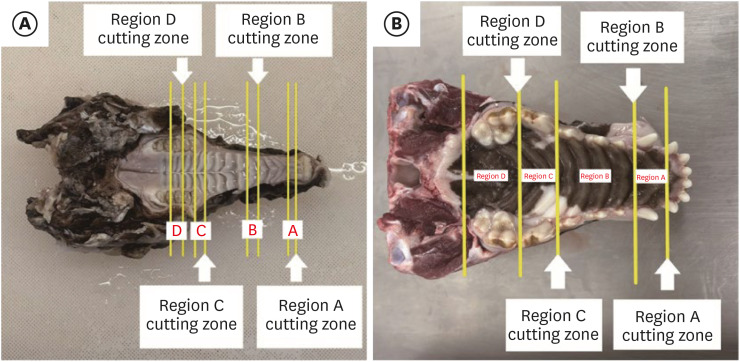
Anatomical segmentation of the nasal cavity into four regions in the rabbit and Beagle. Representative gross images from (A) the rabbit and (B) the Beagle showing division of the nasal cavity into four transverse regions (Regions A–D; anterior, middle, posterior, and olfactory regions) using consistent anatomical landmarks.

For rabbits, the nasal cavities were divided into four transverse regions (A–D; anterior, middle, posterior, and olfactory regions) according to the previously established four-level method [10], which uses dental landmarks including the upper incisors, first palatal ridge, premolars, and molars to obtain reproducible transverse sections. The sampling scheme is illustrated in Fig. 1A.

For Beagles, the nasal cavities were similarly divided into four transverse regions (A–D) using reproducible anatomical landmarks, such as the incisors, canines, molars, and the cribriform plate, thereby reflecting major anatomical differences. This sampling approach was consistent with previous studies [10]. The sampling scheme is illustrated in Fig. 1B.

After removal of the brain and eyeballs, the head was dissected to remove the facial skin, muscles, and mandible, while carefully preserving the external nasal cartilage and cribriform plate.

### Region definitions

For rabbits, Region A (anterior region) was defined posterior to the upper incisor teeth, and Region B (middle region) was posterior to the first palatal ridge. Region C (posterior region) was defined posterior to the first premolar teeth, and Region D (olfactory region) was posterior to the third premolar teeth.

For Beagles, Region A (anterior region) was defined posterior to the last upper incisor teeth, and Region B (middle region) was posterior to the upper canine teeth. Region C (posterior region) was defined posterior to the third premolar teeth, and Region D (olfactory region) was posterior to the fourth premolar teeth.

### Tissue fixation and decalcification

Tissues were fixed in 10% neutral buffered formalin (Duksan Chemical, Korea) for at least 48 h, followed by decalcification in RDO Rapid Decalcifier (APEX Engineering Products, USA) for 48 h.

### Trimming, embedding, and sectioning

Decalcified tissues were trimmed, processed, and embedded in paraffin. Serial sections of 4 μm thickness were prepared using a rotary microtome (Leica RM2235; Leica Biosystems, Germany) and mounted on coated slides.

### Histological staining

Slides were stained with hematoxylin and eosin (H&E; Sigma-Aldrich, USA) using an automated stainer (Tissue-Tek Prisma; Sakura Finetek, Japan). To identify mucin-secreting goblet cells in the nasopharyngeal area of Region B, additional sections were subjected to periodic acid–Schiff (PAS) staining (ScyTek Laboratories, USA) following the manufacturer’s instructions. For Region C in Beagle dogs, only half of the nasal cavity was presented in low-magnification images due to the bilateral symmetry of turbinate structures.

### Histological examination

The histological slides were scanned using a digital slide scanner (Olympus Slideview VS200, Olympus, Japan). Each region was evaluated for epithelial type, the presence and distribution of goblet cells, and the formation of submucosal glands. Five high-power fields were randomly selected from whole slide images of each specific region and structure for the cell counting. Cells were counted and classified in a blinded manner by three different veterinary pathologists. Epithelial cells were classified into five groups: stratified squamous epithelium, transitional epithelium, respiratory epithelium, goblet cells, and olfactory epithelium. Epithelial classification and counting were conducted according to the criteria established through consensus among all three veterinary pathologists. Cell population percentages presented in the figures were calculated as the ratio of the target cell count to the total epithelial cell count (× 100). Epithelial thickness was measured manually using ImageJ software (National Institutes of Health, USA). Comparison of epithelial thickness between rabbits and Beagles was done by measuring each species’ average epithelial thickness. All available animals and histological sections prepared for this study were included in the analysis, and no samples were excluded.

### Statistical analysis

All statistical analyses were performed using GraphPad Prism software (version 9.1; GraphPad Software, USA). Data were presented as mean ± standard deviation. Given the small sample size and the descriptive nature of this study, percentage-based data describing epithelial composition and proportional surface area were presented descriptively and were not subjected to inferential statistical testing. Where quantitative comparisons were retained (epithelial thickness; Supplementary Fig. 1E and F), comparisons between species were performed using unpaired *t*-tests with Welch’s correction on an exploratory basis (two-sided α = 0.05). No formal sample size calculation was performed.

### Artificial intelligence (AI) use disclosure

ChatGPT-5 (OpenAI) was used to refine the clarity of the manuscript. All output was verified by the authors.

## RESULTS

### Comparative gross analysis of the nasal cavity in Beagles and rabbits

**Fig. 2:**
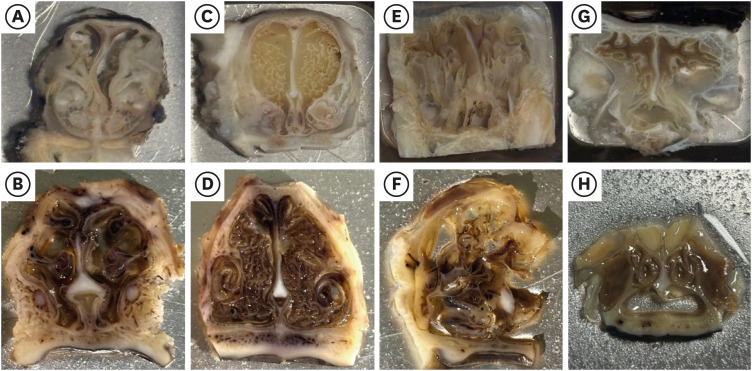
Gross transverse sections of the nasal cavities. (A, B) Transverse section of Region A of (A) the rabbit and (B) the Beagle. (C, D) Transverse section of Region B of (C) the rabbit and (D) the Beagle. (E, F) Transverse section of Region C of (E) the rabbit and (F) the Beagle. (G, H) Transverse section of Region D of (G) the rabbit and (H) the Beagle. Each nasal cavity was divided into four anatomical regions according to consistent landmarks.

In Region A, the rabbit (Fig. 2A) was sectioned posterior to the upper incisor teeth, and the Beagle (Fig. 2B) was cut posterior to the last upper incisor teeth. Region A showed a relatively simple structure compared to the complex structure of the caudal nasal cavity, although the Beagle showed a more complex and thicker structure than the rabbit. In Region B, the rabbit (Fig. 2C) was sectioned posterior to the first palatal ridge, and the Beagle (Fig. 2D) was cut posterior to the upper canine teeth. As Region B reveals the complex structure of nasal turbinates, the Beagle possessed a more twisted and thicker maxilloturbinate than the rabbit. In Region C, the rabbit (Fig. 2E) was sectioned posterior to the first premolar teeth, whereas the Beagle (Fig. 2F) was cut posterior to the third premolar teeth. Proceeding to the transitional zone of respiratory and olfactory function, the structure and space began to narrow. In Region D, the rabbit (Fig. 2G) was sectioned posterior to the third premolar teeth, and the Beagle (Fig. 2H) was sectioned posterior to the fourth premolar teeth. The nasal structure became more simplified than the rostral part of the nasal cavity and its meatus, which continued into the nasopharynx. Upon comparing each region of the rabbit and the Beagle, each corresponding areas were confirmed to represent the same anatomical and histological characteristics of the nasal cavity.

### Comparative histological analysis of the anterior nasal cavity (Region A) in Beagles and rabbits

**Fig. 3:**
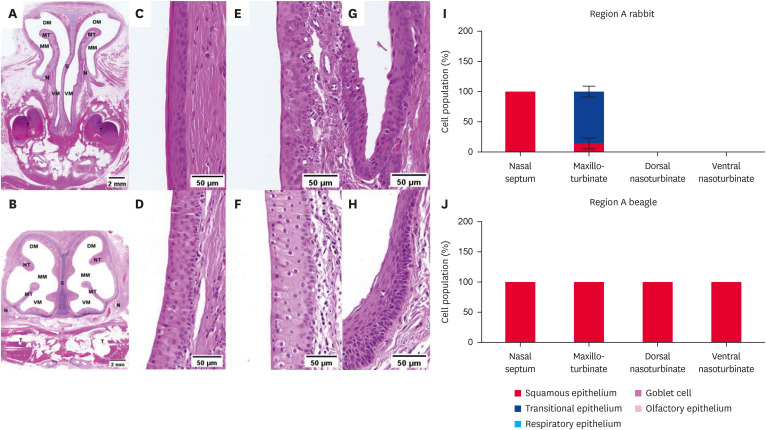
Histological features and quantitative epithelial composition of Region A in the rabbit and Beagle nasal cavity. (A, B) Subgross transverse sections of (A) the rabbit and (B) the Beagle. H&E; scale bar = 2 mm. (C, D) Histological details of the nasal septum in (C) the rabbit lined by squamous epithelium and (D) the Beagle lined by transitional epithelium. (E, F) Histological details of the maxilloturbinate in (E) the rabbit and (F) the Beagle. (G, H) Histological details of the nasolacrimal duct in (G) the rabbit and (H) the Beagle. H&E; scale bar = 50 μm. (I, J) Quantitative classification and composition of epithelium type in (I) the rabbit and (J) the Beagle. H&E, hematoxylin and eosin; DM, dorsal meatus; MM, middle meatus; VM, ventral meatus; MT, maxilloturbinate; NT, nasoturbinate; N, nasolacrimal duct; S, nasal septum; T, tooth.

Both species showed clear segmentation into dorsal, middle, and ventral meatus (Fig. 3A and B). The nasal septum was lined by relatively thin stratified squamous epithelium in rabbits, while it was lined by transitional epithelium in Beagles (Fig. 3C and D). Maxilloturbinate was lined by transitional epithelium in both species, but Beagles was relatively thicker with more prominent submucosal structures (Fig. 3E and F). The nasolacrimal duct was lined by pseudostratified columnar epithelium in both species (Fig. 3G and H). Rabbits showed a mixed distribution of stratified squamous epithelium and transitional epithelium, whereas Beagles were entirely lined by transitional epithelium (Fig. 3I and J).

### Comparative histological analysis of the middle nasal cavity (Region B) in Beagles and rabbits

**Fig. 4:**
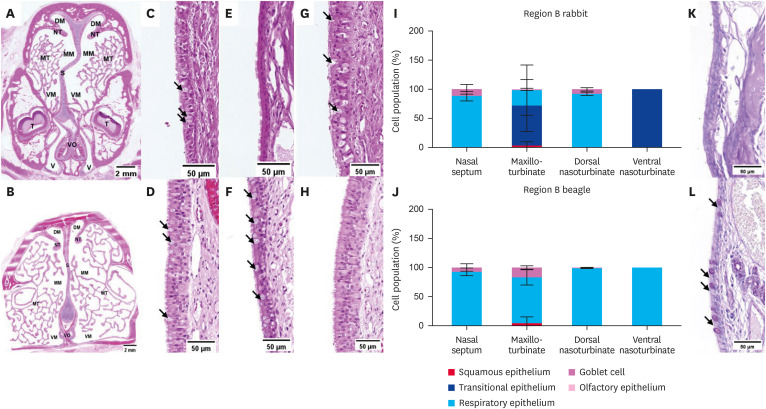
Histological features, quantitative epithelial composition, and PAS staining of Region B in the rabbit and the Beagle nasal cavity. (A, B) Subgross transverse sections of (A) the rabbit and (B) the Beagle. H&E; scale bar = 2 mm. (C, D) Histological details of the nasal septum in (C) the rabbit and (D) the Beagle, both lined by respiratory epithelium. (E, F) Histological details of the maxilloturbinate in (E) the rabbit lined by transitional epithelium and (F) the Beagle lined by respiratory epithelium. (G, H) Histological details of the dorsal nasoturbinate in (G) the rabbit and (H) the Beagle, both lined by respiratory epithelium. H&E; scale bar = 50 μm. (I, J) Quantitative classification and composition of epithelium type in (I) the rabbit and (J) the Beagle. (K, L) PAS staining of maxilloturbinate in (K) the rabbit and (L) the Beagle. PAS stain; scale bar = 50 μm. Arrows indicate the goblet cells. PAS, periodic acid–Schiff; H&E, hematoxylin and eosin; DM, dorsal meatus; MM, middle meatus; VM, ventral meatus; MT, maxilloturbinate; NT, nasoturbinate; N, nasolacrimal duct; S, nasal septum; T, tooth; VO, vomeronasal organ.

Beagles showed a more complex and thicker structure of nasal turbinates than rabbits, including submucosal glands (Fig. 4A and B). Composition of goblet cells in the nasal septum was 12% in rabbits and 7% in Beagles (Fig. 4C and D). In the maxilloturbinate, which consists of the majority of the total surface of the nasal cavity, the goblet cell composition was 1% in rabbits and 16% in Beagles. (Fig. 4E and F). In the dorsal nasoturbinate, the goblet cell composition was 8% in rabbits and 1% in Beagles (Fig. 4G and H). Overall, goblet cells accounted for 3% of epithelial cells in rabbits and 16% in Beagles (Fig. 4I and J). PAS staining further confirmed this species-specific difference, with Beagles showing a broader distribution of PAS-positive goblet cells (Fig. 4K and L).

### Comparative histological analysis of the posterior nasal cavity (Region C) in Beagles and rabbits

**Fig. 5:**
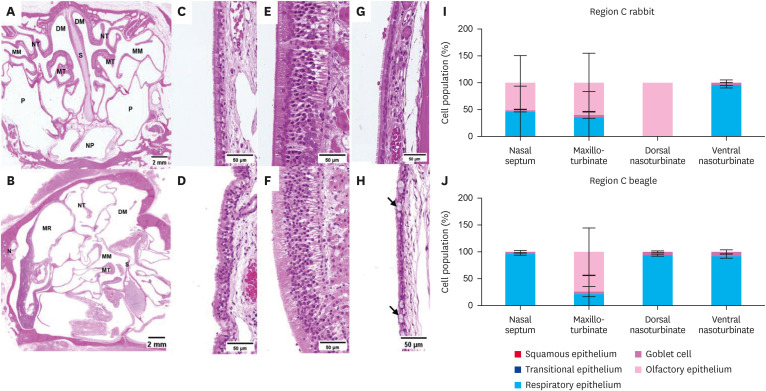
Histological features and quantitative epithelial composition of Region C in the rabbit and Beagle nasal cavity. (A, B) Subgross transverse sections of (A) the rabbit and (B) the Beagle. H&E; scale bar = 2 mm. (C, D) Histological details of the nasal septum in (C) the rabbit lined by both olfactory and respiratory epithelium, and (D) the Beagle lined by respiratory epithelium. (E, F) Histological details of the maxilloturbinate in (E) the rabbit and (F) the Beagle lined by both olfactory and respiratory epithelium. (G, H) Histological details of the ventral nasoturbinate in (G) the rabbit and (H) the Beagle. H&E; scale bar = 50 μm. (I, J) Quantitative classification and composition of epithelium type in (I) the rabbit and (J) the Beagle. Arrows indicate the goblet cells. H&E, hematoxylin and eosin; DM, dorsal meatus; MM, middle meatus; VM, ventral meatus; MT, maxilloturbinate; NT, nasoturbinate; N, nasolacrimal duct; S, nasal septum; NP, nasopharyngeal meatus; P, paranasal sinus; MR, maxillary recess.

The paranasal cavity in rabbits was replaced by the maxillary recess in Beagles, both lined by respiratory epithelium (Fig. 5A and B). In rabbits, the nasal septum showed mixed distribution of olfactory and respiratory epithelium, while it was mostly lined by respiratory epithelium in Beagles (Fig. 5C and D). In the maxilloturbinate, both species showed mixed distribution of epithelial types with a higher proportion of olfactory epithelium than respiratory epithelium (Fig. 5E and F). The ventral nasoturbinate was entirely lined by respiratory epithelium and goblet cells in both species (Fig. 5G-J).

### Comparative histological analysis of the olfactory region (Region D) in Beagles and rabbits

**Fig. 6:**
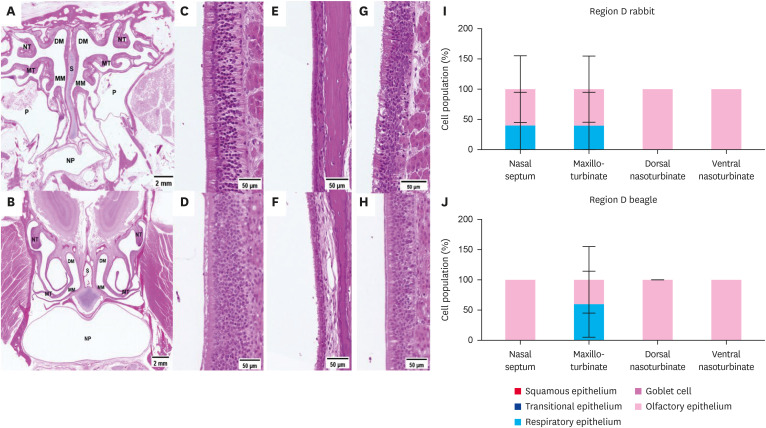
Histological features and quantitative epithelial composition of Region D in the rabbit and Beagle nasal cavity. (A, B) Subgross transverse sections of (A) the rabbit and (B) the Beagle. H&E; scale bar = 2 mm. (C, D) Histological details of the nasal septum in (C) the rabbit lined by both olfactory and respiratory epithelium and (D) the Beagle lined by olfactory epithelium. (E, F) Histological details of the maxilloturbinate in (E) the rabbit and (F) the Beagle lined by both olfactory and respiratory epithelium. (G, H) Histological details of the dorsal nasoturbinate in (G) the rabbit and (H) the Beagle lined by olfactory epithelium. H&E; scale bar = 50 μm. (I, J) Quantitative classification and composition of epithelium type in (I) the rabbit and (J) the Beagle. H&E, hematoxylin and eosin; DM, dorsal meatus; MM, middle meatus; MT, maxilloturbinate; NT, nasoturbinate; S, nasal septum; NP, nasopharyngeal meatus; P, paranasal sinus; MR, maxillary recess.

Both species showed structurally simplified turbinates compared to the rostral regions (Fig. 6A and B). In rabbits, the nasal septum showed mixed distribution of both olfactory and respiratory epithelium, while it was mostly lined by olfactory epithelium in Beagles (Fig. 6C and D). The maxilloturbinate showed a mixed distribution of respiratory and olfactory epithelium in both species (Fig. 6E and F). The surface was entirely lined by olfactory epithelium in nasoturbinates (Fig. 6G and H). Overall, both species showed their surfaces lined mostly by olfactory epithelium, with partial respiratory epithelium in the maxilloturbinate (Fig. 6I and J). The nasopharyngeal meatus was lined with respiratory epithelium in both species.

### Quantitative comparison of epithelium composition and epithelial thickness in Beagles and rabbits

Rabbits and Beagles showed a difference in the proportional surface area of anatomical structures of the nasal cavity. Beagles showed a tendency to possess a higher proportion of turbinate surface, especially maxilloturbinate, compared to rabbits (Supplementary Fig. 1A and B). In Region A, rabbits showed a mixed appearance of stratified squamous epithelium (50%) and transitional epithelium (50%), while Beagles were entirely lined by transitional epithelium. In Region B, rabbits consisted of transitional epithelium (55%) and respiratory epithelium (39%) with sparse goblet cells (3%), whereas Beagles consisted of mainly respiratory epithelium (79%) with abundant goblet cells (16%). In Regions C and D, only minor differences were shown, with olfactory epithelium as a dominant epithelium type, followed by respiratory epithelium (Supplementary Fig. 1C and D). The thickness of the respiratory epithelium was almost even in both species (Supplementary Fig. 1E). However, the olfactory epithelium of Beagles was much thicker than that of rabbits (Supplementary Fig. 1F).

## DISCUSSION

The nasal turbinate is a structurally complex organ that plays a critical role in conditioning inspired air and providing the first line of defense against inhaled toxicants. Despite this importance, region-specific histological criteria for Beagle nasal turbinates have not been fully standardized, leading to inconsistent toxicological and pathological evaluations. One study conducted a toxicopathological evaluation in Beagles by dividing the nasal cavity into four transverse sections, but the rationale for the anatomical landmarks used to define these sections was insufficiently described [16]. This study addressed this gap by comparing the nasal turbinates of Beagles and rabbits, defining region-specific histological features, and proposing reproducible criteria for inhalation toxicity studies.

In rabbits, histological evaluation of the nasal cavity is well established, with subdivision into anterior, middle, posterior, and olfactory regions that enables lesion localization and inference of toxicity severity [17]. The anterior, middle, and posterior nasal segments are primarily lined by respiratory epithelium rich in goblet cells, so the severity and time course of injury can be judged by examining epithelial metaplasia, hyperplasia, and fibrosis [17,18]. Notably, lesions typically appear earlier and more severely in the anterior nasal cavity, allowing semi-quantitative assessment of epithelial toxicity. The olfactory region provides additional functional endpoints, including loss of olfactory epithelium, alterations in Bowman’s glands, and damage to olfactory nerve bundles [17,19]. However, this scheme contains transition zones between nasal subregions; these boundaries are relatively narrow in the rabbit, so even slight variation in trimming planes can alter the proportion of mixed epithelia and compromise quantitative interpretation. In addition, the thin bony turbinates of the rabbit make rabbit specimens highly sensitive to minor deviations in sectioning planes, reducing reproducibility.

The Beagle nasal cavity exhibited a few distinct features. Gross findings confirmed that Beagles have a long nasal cavity with thick, complex turbinates, which provide clear regional landmarks and enable consistent orientation and sectioning. In addition, Beagles possess an extensive olfactory region with fine structural partitioning, making them suitable for detecting toxic injury to the olfactory epithelium and olfactory nerves [20]. Meanwhile, it has also been reported that the nasal epithelial composition of the dog is more similar to that of humans than to that of rabbits or rodents [21]. Overall, these distinct traits of the Beagle nasal cavity reflect not only its advantages as an inhalation toxicity model but also the potential for translational research. Thus, the necessity for species-specific histological reference standards for canine inhalation toxicity studies was highlighted [22].

The transverse segmentation into four regions (A–D) proved effective for the systematic evaluation of epithelial distribution and mucosal architecture in both species. Our gross anatomical findings showed that Beagles have elongated nasal cavities and complex turbinates, providing a large mucosal surface for deposition of inhaled toxicants. Quantitative analysis of this study revealed a broad distribution of respiratory epithelium and thick olfactory epithelium, providing histological data of the Beagle nasal cavity as a model for inhalation studies. Moreover, it revealed differences in epithelial type and goblet cell distribution among nasal cavity structures in rabbits and Beagles across the four regions. These features highlight the Beagle’s suitability as a non-rodent model for inhalation studies, consistent with previous recommendations from regulatory studies. Region-specific histological evaluation showed reproducible epithelial patterns aligned with the functional divisions of the nasal cavity [23]. Histopathological characteristics of each specific region and their toxicopathological implications are summarized in Supplementary Table 1. These findings support the use of transverse anatomical landmarks as reliable reference points for histological standardization.

Region A, functionally, serves as the anterior entry zone of the nasal cavity, mainly involved in air intake and primary filtration, with its epithelial organization providing the first line of mucosal defense against inhaled agents [24]. In Region A, while stratified squamous and transitional epithelium each account for nearly half of the composition in rabbits, Beagles are entirely lined in transitional epithelium. Stratified squamous epithelium lacks the innate immune function of a physical barrier. On the other hand, the transitional epithelium is a transitional form to the respiratory epithelium in the nasal cavity, lined by pseudostratified columnar epithelium with a limited population of cilia and goblet cells. Despite its limited capacity, it contributes to innate immunity through mechanical clearance. These findings imply that Beagles, whose transitional epithelium is distributed more rostrally, may possess more extensive mechanical clearance capacity than rabbits in Region A. Region B serves as the core zone for mucus secretion and air purification, characterized by turbulent airflow with active mucus secretion that enhances filtration and humidification of inhaled air [11]. In Region B, whereas rabbits have a majority of their surface lined by transitional epithelium, Beagles are mostly lined by respiratory epithelium with more abundant goblet cells. In both species, Region B has the highest percentage of the maxilloturbinate surface area and the greatest proportions of respiratory epithelium and goblet cells among the four regions. This implies that Region B plays a central role in the innate defense mechanism of the nasal cavity. It has been known that the complexity of the nasal turbinates contributes to the efficiency of particle capture in inhaled air [25]. Compared to rabbits, the Beagle exhibits a higher proportional surface area of the maxilloturbinate, which contributes majorly to the complexity of the nasal cavity structure. Furthermore, the respiratory epithelium and the goblet cell population are mainly concentrated in the maxilloturbinate. Region C serves as a transitional zone that combines airflow and mucus clearance with olfactory signal transmission, while Region D is specialized for olfactory detection and neural signal transduction, supported by dense olfactory epithelium that contributes to odor sensing [26]. Respiratory and olfactory epithelium cooperate to maintain effective mucociliary transport and odor detection [27]. In Regions C and D, a major proportion of the surface is lined by olfactory epithelium in both species. Thus, it can be speculated that these regions are the core for the olfactory function. The olfactory epithelium of the Beagle was histologically confirmed to be more structurally developed and measured to be thicker than that of the rabbit. These histological traits of the Beagle may be identified as distinct features in the histopathological evaluation of inhalation toxicity studies.

PAS staining revealed distinct differences in goblet cell distribution between Beagles and rabbits. In response to early external irritation and inflammation in the turbinates, goblet cells initially undergo hyperplasia and increased mucus secretion, thereby diluting inhaled toxicants and promoting their clearance. As exposure becomes chronic, persistent stimulation leads to epithelial metaplasia, glandular hypertrophy, and mucus retention, thereby increasing the risk of secondary infection [28,29]. Beagles exhibited broader mucin-secreting goblet cells, which may suggest a more extensive mucosal defense capacity compared with rabbits. These findings suggest that Beagle may better represent mucin-related responses to inhaled toxicants, providing translational relevance for human risk assessment. Goblet cell quantification further supported the utility of PAS staining as an indicator of mucosal defense integrity.

Together, these findings establish standardized histological landmarks for Beagle nasal turbinates, filling a critical gap in comparative toxicology. Even though rabbit-based frameworks have long guided region-specific evaluations [10,17], the lack of Beagle-specific criteria has limited interspecies comparisons and undermined reproducibility. Our results provide the first standardized, region-based reference for Beagle turbinates, enabling more accurate lesion interpretation, and facilitate cross-species extrapolation. Such standardization is crucial for enhancing reproducibility across laboratories, improving accuracy in pathological evaluation, and supporting regulatory requirements for inhalation toxicity testing. Moreover, the framework proposed here will aid in distinguishing toxicant-induced lesions from normal anatomical variations, thereby reducing interpretative errors in safety assessments.

Beagle’s traits of nasal epithelium enable extensive and accurate histopathological diagnosis in toxicity studies. The Beagle nasal cavity is entirely lined by transitional, respiratory, and olfactory epithelium. This feature enables evaluation of toxicant effects and epithelial changes throughout the nasal cavity. Also, well-separated layers of olfactory epithelium enable precise analysis of each cell type, which is critical in histopathological toxicity evaluation. Moreover, the olfactory epithelium is unique among olfactory sensory neurons, as it is in direct contact with the external environment. It has been shown that olfactory sensory neurons are damaged by inhaled chemicals and pathogens, thereby impairing olfactory capacity [30]. Thus, the importance of olfactory sensory neurons in inhalation and neural toxicity research is being highlighted. The elaborate olfactory epithelium of the Beagle may further support its potential as a model for future neurotoxicity studies.

This study has several limitations. First, the sample size was relatively small, which limits the statistical power of inferential analyses. Second, percentage-based data describing epithelial composition and proportional surface area are inherently bounded and should be interpreted cautiously. Accordingly, the present findings are intended to describe comparative anatomical trends rather than to support definitive inferential conclusions. Third, the sex of the animals used in the study varies between the species. Although it has been reported that there is no significant gender difference in New Zealand White rabbits, it would have been preferable to consider variation within the species [31,32,33]. Meanwhile, the Beagles were limited to females to control for potential gender differences, since this subject is not clearly defined. As the criteria in this study are provided as a preliminary reference framework, further related studies with larger cohorts, different sexes and ages, and multiple laboratories should be conducted and reviewed to clearly validate the findings in this study.

In conclusion, this study provides standardized, region-specific histological criteria for Beagle nasal turbinates, producing a reproducible anatomic reference. By demonstrating consistent landmarks and quantifying the epithelial distribution in four transverse regions, this study presents unique histological traits of the Beagle. These standardized criteria provide essential baseline data for toxicologic pathology, improve reproducibility across laboratories, and advance the translational reliability of animal-to-human extrapolation in respiratory toxicology.

## SUPPLEMENTARY MATERIALS

Supplementary Table 1 Histological features and pathological implications in Beagle nasal regions (A–D)

Supplementary Fig. 1 Quantitative analysis and comparison of nasal cavity epithelium composition and epithelial thickness in the rabbit and Beagle. (A, B) Region-specific proportional surface area of the nasal cavity structures in (A) the rabbit and (B) the Beagle. (C, D) Region-specific epithelium classification in (C) the rabbit and (D) the Beagle. (E) Comparison of respiratory epithelial thickness. (F) Comparison of olfactory epithelial thickness.
